# Glycosylation of a Newly Functionalized Orthoester Derivative

**DOI:** 10.3390/molecules19022602

**Published:** 2014-02-24

**Authors:** Kohei Kawa, Tsuyoshi Saitoh, Eisuke Kaji, Shigeru Nishiyama

**Affiliations:** Department of Chemistry, Faculty of Science and Technology, Keio University, Hiyoshi 3-14-1, Kohoku-ku, Yokohama 223-8522, Japan

**Keywords:** glycosylation, orthoester, electrogenerated acid (EGA), Fmoc group, selective deprotection

## Abstract

Tandem glycosylation of the 6-*O*-Fmoc-substituted benzyl orthoester derivative **2a** was carried out in moderate yields by electrogenerated acid (EGA). The Fmoc group was effectively removed under mild basic conditions, and the product was submitted to the subsequent glycosylation.

## 1. Introduction

**Figure 1:**
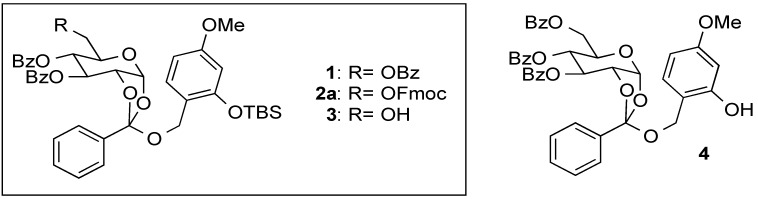
The benzyl orthoesters **1**–**4**.

With the discovery of the numerous biologically important roles of sugar chains, a number of glycosylation protocols have been reported [1,2]. Generally, the glycosylation process consists of efficient generation of an oxocarbenium ion species as a glycosyl donor and modulation of its selective nucleophilic attack on aglycons. Enhancement of β-selectivity is supported by the participation of acyl-protected hydroxyl groups. In particular, construction of a cyclic carboxonium ion between the C1 and C2 positions is an effective method to obtain the corresponding β-glycosidic linkage of D-sugars, although orthoesters are produced as side products when sterically hindered functional groups are used in glycosyl donors and acceptors [3,4,5,6]. Orthoesters were known to be converted to the corresponding glycosides under acidic conditions, and relatively stable derivatives were employed as glycoside precursors by Kochetkov [7,8,9,10,11] and Kunz [12,13]. We reported the synthesis and reaction of the orthoester **1** (Figure 1), which could be purified by silica gel column chromatography and kept for one month at room temperature [14]. Upon glycosylation with appropriate alcohols in the presence of the electrogenerated acid (EGA) [15,16,17,18,19,20], which is considered to be anhydrous HClO_4_ produced by anodic oxidation of cyclohexanol and Bu_4_NClO_4_, the corresponding glycosides were produced in high (primary OH) to moderate (secondary OH) yields, even on tertiary OH groups [14]. Upon comparison with Lewis acids and Brønsted acids, EGA provided better results in glycosylation reactions. To synthesize sugar chains by repeated glycosylation, devices providing effective activation of the anomeric center and the subsequent selective deprotection of an appropriate hydroxyl group in sugar units are required. The orthoester **1** was modified to examine its applicability to practical tandem glycosylation. Here, we describe a synthesis of the orthoester derivative **2a** carrying an Fmoc group at the C-6 position, and its glycosylation reaction.

## 2. Results and Discussion

To examine the chemical properties of the orthoester **1** for the modulation of protecting groups, the acyl protecting groups were removed under basic conditions to give an unstable triol. Although removal of the TBS group in the benzylic region provided the stable phenol derivative **4** [14], the triol was labile under standard work-up conditions, and only detected by ESI mass spectrum. These observations prompted us to synthesize the orthoester derivative **2a**, in which a protecting group at the C-6 position can be selectively removed. After assessment of various protecting groups, we adopted the Fmoc group, which is removed under mild basic conditions.

### 2.1. Synthesis of the Orthoester **2a**

**Scheme 1:**
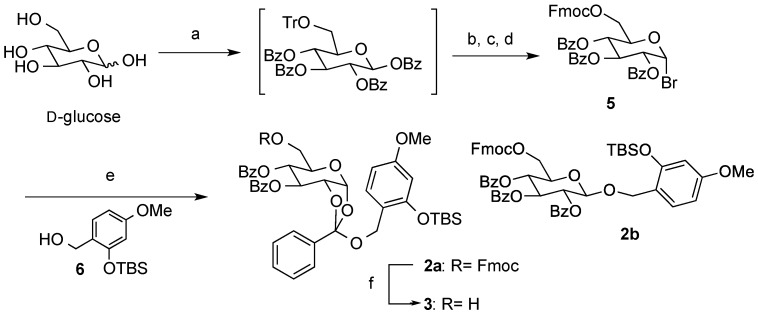
Synthesis of the orthoester derivative **2a**.

Synthesis of the C-6 modified orthoester **2a** commenced with conversion of 6-*O*-trityl-1,2,3,4-tetra-*O*-benzoyl-β-d-glucopyranose, which was synthesized by the standard procedure from D-glucose, into 6-*O*-Fmoc-2,3,4-tri-*O*-benzoyl-α-d-glucopyranosyl bromide (**5**) using a two-step manipulation, followed by the anomeric bromination (Scheme 1). Glycosylation of **5** with the benzyl alcohol **6** under Königs–Knorr reaction conditions provided the expected orthoester **2a** (50%) [21], along with the corresponding benzyl glycoside **2b** (30%). Stability of **2a** under standard work-up and chromatographic conditions was similar to the previously reported derivative **1**. The Fmoc group in **2a** was selectively removed under basic conditions to give 6-OH orthoester **3**, which will be used as a glycosyl acceptor under neutral glycosylation conditions.

*Reagents and yields:* (a) TrCl, Et_3_N, pyr.; BzCl. (b) H_2_Pd/C. (c) FmocCl, pyr. (d) HBr-AcOH, Ac_2_O, AcOH (43% in four steps. (e) **6**, Hg(CN)_2_, 4Å MS (**2a**: 50%, **2b**: 30%). (f) pyr. (81%).

### 2.2. Glycosylation of the Orthoester **2a**

**Scheme 2:**
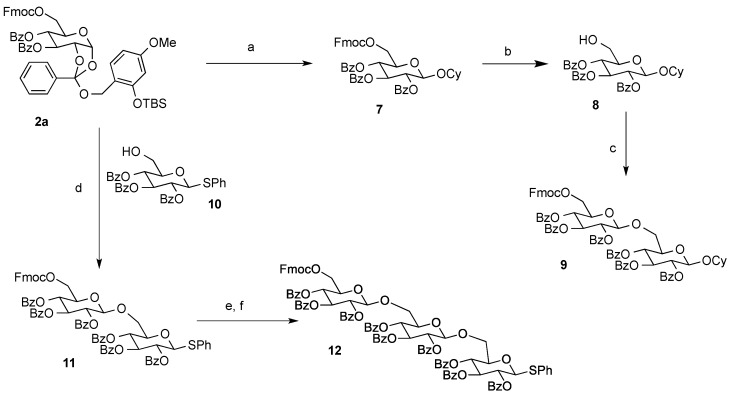
Glycosylation reactions of the orthoester derivative **2a**.

When the orthoester **2a** was submitted to reaction with cyclohexanol in the presence of EGA [14] and 4Å MS in 1,2-DCM at 40 °C, the expected β-glycoside **7** was obtained in 62% yield (Scheme 2). Next selective removal of the Fmoc group in **7** under basic conditions was carried out to give **8** in 95% yield, without any acyl migration from C-4 to C-6 position or removal of the acyl group. Repeated glycosylation of **8** with **2a** under the same conditions as mentioned above, gave the disaccharide **9** in 38% yield. Alternatively, glycosylation of **10** with **2a** provided the disaccharide **11** in 53% yield. After removal of the Fmoc group, further glycosylation with **2a** provided the corresponding trisaccharide **12** [22].

*Reagents and yields*: (a) cyclohexanol, EGA, 4Å MS (62%). (b) Et_3_N, pyr. (95%). (c) **2a**, EGA, 4Å MS (38%). (d) **10**, EGA, 4Å MS (63%). (e) Et_3_N, pyr. (82%). (f) **2a**, EGA, 4Å MS (12%).

## 3. Experimental

### General Information

All reactions were carried out under an argon atmosphere unless otherwise noted. When necessary, solvents were dried prior to use. Dry THF, dry Et_2_O and dry CH_2_Cl_2_ were purchased from Kanto Chemical Co., Inc. (Tokyo, Japan). Optical rotations were measured on a JASCO P-2200 digital polarimeter with a sodium (D line) lamp. IR spectra were recorded on a JASCO Model A-202 spectrophotometer. ^1^H-NMR spectra and ^13^C-NMR spectra were obtained on JEOL JNM-GX400, JNM-α400, JNM-AL400 or JNM-ECX400 spectrometers in deuterated solvent using tetramethylsilane as an internal standard. Deuteriochloroform was used as a solvent, unless otherwise stated. High-resolution mass spectra were obtained on a Waters LCT Premier XE (ESI) or JEOL JMS-700 (FAB). Preparative and analytical TLC were carried out on silica gel plate (Kieselgel 60 F254, E. Merck AG., Darmstadt, Germany) using UV light, 1 M aq. sulfuric acid, and/or 5% molybdophosphoric acid in ethanol for detection. Kanto silica 60N (spherical, neutral, 105–210 μm) was used for column chromatography. All anodic oxidation was conducted using HA-151A (Hokuto Denko, Tokyo, Japan) as a potentiostat and galvanostat, using glassy carbon plate as anodes, and platinum plate or wire as cathode.

*2,3,4-Tri-O-benzoyl-6-O-9-fluorenylmethyloxycarboxyl-α-D-glucopyranosyl bromide* (**5**)*.* To a suspension of d-glucose (4.55 g, 0.025 mol) in pyridine (50 mL) were added TrCl (10.6 g, 0.038 mol) and Et_3_N (18 mL, 0.13 mol), and the mixture was stirred at room temperature overnight. After the addition of BzCl (23 mL, 0.20 mol) at the same temperature, stirring was continued overnight. The reaction was quenched with sat. aq. NaHCO_3_, and the mixture was extracted with CHCl_3_. The organic layer was dried (Na_2_SO_4_), and concentrated *in* *vacuo*. After removal of the high polarity byproducts by silica gel short column (hexane/EtOAc = 5:1), a crude was used in the next step without further purification. The crude product was solved in MeOH/EtOAc (1:1, 100 mL). After the addition of 5% Pd-C (cat.), the mixture was stirred overnight under H_2_ atmosphere. The mixture was filtered and the filtrate was concentrated *in* *vacuo*. After silica gel short column chromatography (hexane/EtOAc = 3:1), a crude was used in the next step without further purification.

A mixture of the crude and FmocCl (9.80 g, 0.038 mol) in pyridine (100 mL) was stirred overnight. The reaction was quenched by sat. aq. NaHCO_3_, and the mixture was extracted with CHCl_3_. The organic layer was dried (Na_2_SO_4_), and concentrated *in* *vacuo*. After purification by silica gel short column chromatography (hexane/EtOAc = 10:1), a crude was used in the next step without further purification. A mixture of the crude, HBr-AcOH (250 mL), AcOH (60 mL), and Ac_2_O (60 mL) was stirred overnight. The mixture was extracted with CHCl_3_. The organic layer was dried (Na_2_SO_4_), and concentrated *in* *vacuo*. The residue was purified by silica gel column chromatography (hexane/EtOAc = 5:1) to give **5** as a colorless oil (8.43 g, 43% in 4 steps): [α]^23^_D_ +76.4 (*c* 1.00, CHCl_3_); IR (film) 1732, 1601, 1261 cm^−1^; ^1^H-NMR (400 MHz) δ 4.40 (5H, m, H-6a,6b, Fmoc), 4.67 (1H, m, H-5), 5.36 (1H, dd, *J* = 9.8, 4.0 Hz, H-2), 5.78 (1H, t, *J* = 9.8 Hz, H-4), 6.28 (1H, t, *J* = 9.8 Hz, H-3), 6.89 (1H, d, *J* = 4.0 Hz, H-4), 7.40 (11H, m, Ar), 7.54 (2H, m, Ar), 7.64 (2H, m, Ar), 7.78 (2H, m, Ar), 7.89 (2H, m, Ar), 8.00 (4H, m, Ar); ^13^C-NMR (100 MHz) δ 46.5 (Fmoc), 64.9 (C-6), 67.7 (C-4), 70.4 (C-3), 70.5 (C-2), 71.3 (C-5), 72.5 (FmocCH_2_), 86.7 (C-1), 120.0 (Ar), 125.2 (Ar), 125.3 (Ar), 127.2 (Ar), 127.9 (Ar), 128.3 (Ar), 128.4 (Ar), 128.5 (Ar), 128.7 (Ar), 129.7 (Ar), 129.9 (Ar), 130.0 (Ar), 133.3 (Ar), 133.7 (Ar), 133.85 (Ar), 141.2 (Ar), 141.3 (Ar), 143.2 (Ar), 143.4 (Ar), 154.7 (C=O), 165.1 (C=O), 165.2 (C=O), 165.5 (C=O). ESI-MS: calcd for C_42_H_33_O_10_NaBr 799.1155 (M+Na)^+^, found *m/z* 799.1151.

*(2R,5R,6R,7S)-5-(((((9H-Fluoren-9-yl)methoxy)carbonyl)oxy)methyl)-2-((2-((tert-butyldimethylsilyl)oxy)-4-methoxybenzyl)oxy)-2-phenyltetrahydro-3aH-[1,3]dioxolo[4,5-b]pyran-6,7-diyl dibenzoate* (**2a**) and * 2-tert-Buthyldimethylsyloxy-4-methoxyphenylmethyl2,3,4-tetra-O-benzoyl-6-O-9-fluorenylmethyloxycarboxyl-β-d-glucopyranoside* (**2b**). To a solution of **5** (71 mg, 0.092 mmol) in anhydrous toluene (0.9 mL) were added **6** (74 mg, 0.27 mmol), Hg(CN)_2_ (46 mg, 0.18 mmol), and MS 4A at 100 °C, and the mixture was stirred overnight. The mixture was filtered through a Celite pad, and the filtrate was washed with sat. aq. NaHCO_3_. The organic extracts were dried (Na_2_SO_4_), and concentrated *in vacuo*. The residue was purified by silica gel column chromatography (hexane/acetone = 7:1) to give **2a** (45 mg, 50%) and **2b** **(**26 mg, 30%) as oil: **2a**: [α]^23^_D_ +1.2 (c 1.00, CHCl_3_); IR (film) 1728, 1613, 1257 cm^−1^; ^1^H-NMR (400 MHz) δ 0.15 (6H, s, SiCH_3_), 0.93 (9H, s, tBu), 3.74 (3H, s, OCH_3_), 4.16 (2H, m, Fmoc), 4.29 (6H, m, H-5,6a,6b, ArCH_2_, Fmoc), 4.78 (1H, m, H-2), 5.37 (1H, bd, H-4), 5.76 (1H, br, H-3), 6.04 (1H, bd, H-1), 6.31 (1H, d, *J* = 2.2 Hz, Ar), 6.48 (1H, dd, *J* = 8.3, 2.2 Hz, Ar), 7.18 (1H, d, *J* = 8.3 Hz, Ar), 7.41 (11H, m, Ar), 7.57 (4H, m, Ar), 7.75 (2H, m, Ar), 7.82 (2H, m, Ar), 7.97 (2H, m, Ar), 8.11 (2H, m, Ar); ^13^C-NMR (100 MHz) δ −4.3 (SiCH_3_), 18.1 (tBu), 25.6 (tBu), 46.6 (Fmoc), 55.2 (OCH_3_), 61.3 (ArCH_2_), 67.3 (C-6), 68.3 (C-2), 69.2 (C-3), 70.1 (C-4), 72.3 (FmocCH_2_), 77.2 (C-5), 97.4 (C-1), 105.3 (Ar), 105.9 (Ar), 119.9 (Ar), 120.5 (Ar), 121.4 (Ar), 125.3 (orthoester-C), 126.5 (Ar), 127.1 (Ar), 127.2 (Ar), 127.8 (Ar), 128.3 (Ar), 1283.4 (Ar), 128.5 (Ar), 129.1 (Ar), 129.6 (Ar), 129.9 (Ar), 130.0 (Ar), 133.5 (Ar), 133.6 (Ar), 135.3 (Ar), 141.2 (Ar), 143.2 (Ar), 154.0 (C=O), 154.9 (C=O), 159.9 (C=O), 165.2 (C=O). ESI-MS: calcd for C_56_H_56_O_13_SiNa 987.3388 (M+Na)^+^, found *m/z* 987.3373. **2b**: [α]^23^_D_ −14.9 (c 1.00, CHCl_3_); IR (film) 1735, 1610, 1262 cm^−1^; ^1^H-NMR (400 MHz) δ 0.10 (3H, s, SiCH_3_), 0.16 (3H, s, SiCH_3_), 0.94 (9H, s, tBu), 3.72 (3H, s, OCH_3_), 4.06 (1H, ddd, *J* = 9.8, 5.3, 3.2 Hz, H-5), 4.23 (1H, t, *J* = 7.6 Hz, Fmoc), 4.40 (4H, m, H-6a,6b, Fmoc), 4.76 (1H, d, *J* = 12.4 Hz, ArCH_2_), 4.83 (1H, d, *J* = 12.4 Hz, ArCH_2_), 4.86 (1H, d, *J* = 8.0 Hz, H-1), 5.58 (1H, dd, *J* = 9.8, 8.0 Hz, H-2), 5.60 (1H, t, *J* = 9.8 Hz,H-4), 5.83 (1H, t, *J* = 9.8 Hz, H-3), 6.27 (1H, dd, *J* = 8.7, 2.5 Hz, Ar), 6.30 (1H, d, *J* = 2.5 Hz, Ar), 7.12 (1H, d, *J* = 8.7 Hz, Ar), 7.35 (14H, m, Ar), 7.50 (2H, m, Ar), 7.62 (2H, m, Ar), 7.76 (2H, m, Ar), 7.83 (4H, m, Ar), 7.92 (1H, m, Ar); ^13^C-NMR (100 MHz) δ −4.4 (SiCH_3_), −4.3 (SiCH_3_), 18.1 (tBu), 25.6 (tBu), 29.7 (tBu), 46.6 (Fmoc), 55.2 OCH_3_), 65.6 (ArCH_2_), 66.3 (C-6), 69.6 (C-4), 70.2 (C-3), 71.6 (C-2), 72.0 (C-5), 72.9 (FmocCH_2_), 99.1 (C-1), 105.4 (Ar), 105.9 (Ar), 119.6 (Ar), 119.9 (Ar), 125.2 (Ar), 125.3 (Ar), 127.2 (Ar), 127.8 (Ar), 128.1 (Ar), 128.2 (Ar), 128.4 (Ar), 128.8 (Ar), 129.3 (Ar), 129.7 (Ar), 129.8 (Ar), 129.9 (Ar), 131.2 (Ar), 133.0 (Ar), 133.2 (Ar), 133.4 (Ar), 141.2 (Ar), 143.2 (Ar), 143.4 (Ar), 154.7 (C=O), 154.9 (C=O), 160.2 (C=O), 164.9 (C=O), 165.2 (C=O), 165.8 (C=O). ESI-MS: calcd for C_56_H_56_O_13_SiNa 987.3388 (M+Na)^+^, found *m/z* 987.3381.

*(2R,5R,6R,7S)-2-((2-((tert-Butyldimethylsilyl)oxy)-4-methoxybenzyl)oxy)-5-(hydroxymethyl)-2-phenyl-tetrahydro-3aH-[1,3]dioxolo[4,5-b]pyran-6,7-diyl dibenzoate* (**3**). To a solution of **2a** (30 mg, 0.032 mmol) in pyridine (0.5 mL) was added Et_3_N (13 µL, 0.095 mmol) at room temperature. After being stirred overnight, the reaction mixture was concentrated *in vacuo*. The residue was purified by silica gel column chromatography (hexane/acetone = 2:1) to give **3** as a clear oil (19 mg, 81%): [α]^23^_D_ +9.5 (c 1.00, CHCl_3_); IR (film) 3527, 1725, 1613, 1261 cm^−1^; ^1^H-NMR (400 MHz) δ 0.15 (6H, s, SiCH_3_), 0.90 (9H, s, tBu), 3.65 (1H, m, H-6), 3.75 (3H, s, OCH_3_), 3.78 (1H, m, H-6'), 3.89 (1H, m, H-5),4.29 (1H, d, *J* = 11.2 Hz, ArCH_2_), 4.36 (1H, d, *J* = 11.2 Hz, ArCH_2_), 4.75 (1H, ddd, *J* = 5.2, 2.9, 0.8 Hz, H-2), 5.37 (1H, m, H-4), 5.74 (1H, m, H-3), 6.03 (1H, bd, H-1), 6.31 (1H, d, *J* = 2.5 Hz, Ar), 6.48 (1H, dd, *J* = 8.5, 2.5 Hz, Ar), 7.18 (1H, d, *J* = 8.5 Hz, Ar), 7.44 (7H, m, Ar), 7.60 (2H, m, Ar), 7.81 (2H, m, Ar), 7.98 (2H, m, Ar), 8.05 (2H, m, Ar); ^13^C-NMR (100 MHz) δ −4.3 (SiCH_3_), 18.1 (tBu), 25.6 (tBu), 55.2 (OCH_3_), 61.2 (ArCH_2_), 62.4 (C-6), 68.3 (C-2), 69.4 (C-3), 69.8 (C-4), 72.6 (C-5), 97.5 (C-1), 105.3 (Ar), 105.9 (Ar), 120.5 (Ar), 121.1 (Ar), 126.4 (orthoester-C), 128.3 (Ar), 128.4 (Ar), 128.5 (Ar), 129.0 (Ar), 129.1 (Ar), 129.6 (Ar), 129.9 (Ar), 130.0 (Ar), 133.5 (Ar), 133.6 (Ar), 135.3 (Ar), 154.0 (C=O), 159.9 (C=O), 164.6 (C=O), 165.6 (C=O). ESI-MS: calcd for C_41_H_46_O_11_NaSi 765.2707 (M+Na)^+^, found *m/z* 765.2710.

*Cyclohexyl 2,3,4-tri-O-benzoyl-6-O-9-fluorenylmethyloxycarboxyl-β-d-glucopyranoside* (**7**). A 0.1 M solution of cyclohexanol in 1,2-DCE (10 mL) containing 4Å MS and Bu_4_NClO_4_ (3.42 g, 1 M) as a supporting salt, was electrolyzed by constant current electrolysis (C.C.E., 6 mA/cm^2^) at 40 °C, using a glassy carbon plate (1.5 cm × 1.5 cm) as an anode and a Pt plate (1.8 cm × 1.8 cm) as a cathode. After the electrolysis, the reaction mixture (3.4 mL, 0.1 M EGA solution, 3 equiv.) was added to a solution of **2a** (0.11 g, 0.11 mmol), cyclohexanol (0.4 mL, 0.34 mmol), and 4Å MS in 1,2-DCE (1.1 mL). After being stirred 20 min, the mixture was filtered through a Celite pad and the filtrate was washed with sat. aq. NaHCO_3_. The organic extracts were dried (Na_2_SO_4_), and concentrated *in vacuo*. The residue was purified by preparative TLC (hexane/acetone = 5:1) to give **7** as an oil (0.056 g, 62%): [α]^23^_D_ −4.10 (*c* 1.00, CHCl_3_); IR (film) 1732, 1601, 1262 cm^−1^; ^1^H-NMR (400 MHz) δ 1.22 (5H, m, cyclohexyl), 1.46 (2H, m, cyclohexyl), 1.70 (2H, m, cyclohexyl), 1.88 (1H, m, cyclohexyl), 3.72 (1H, m, cyclohexyl), 4.09 (1H, m, Fmoc), 4.22 (1H, m, Fmoc), 4.36 (3H, m, H-5,6a, Fmoc), 4.46 (1H, d, *J* = 11.4, 6.5 Hz, H-6b), 4.93 (1H, d, *J* = 8.0 Hz, H-1), 5.51 (1H, dd, *J* = 9.6, 8.0 Hz, H-2), 5.55 (1H, t, *J* = 9.6 Hz, H-4), 5.90 (1H, t, *J* = 9.6 Hz, H-3), 7.30 (11H, m, Ar), 7.51 (2H, m, Ar), 7.60 (2H, m, Ar), 7.77 (2H, m, Ar), 7.84 (2H, m, Ar), 7.95 (4H, m, Ar); ^13^C-NMR (100 MHz) δ 23.4 (cyclohexyl), 23.6 (cyclohexyl), 25.3 (cyclohexyl), 31.5 (cyclohexyl), 33.1 (cyclohexyl), 46.6 (Fmoc), 66.5 (C-6), 69.8 (C-4), 70.2 (C-2,3), 72.0 (FmocCH_2_), 72.9 (C-5), 78.2 (cyclohexyl), 99.7 (C-1), 120.0 (Ar), 125.2 (Ar), 127.2 (Ar), 127.9 (Ar), 128.3 (Ar), 128.4 (Ar), 128.7 (Ar), 128.8 (Ar), 129.5 (Ar), 129.7 (Ar), 129.8 (Ar), 133.1 (Ar), 133.2 (Ar), 133.5 (Ar), 141.2 (Ar), 143.2 (Ar), 123.3 (Ar), 154.8 (C=O), 164.9 (C=O), 165.3 (C=O), 165.8 (C=O). ESI-MS: calcd for C_48_H_45_O_11_7997.2962 (M+H)^+^, found *m/z* 797.2936.

*Cyclohexyl 2,3,4-tri-O-benzoyl-β-d-glucopyranoside* (**8**). To a solution of **7** (54 mg, 0.068 mmol) in pyridine (1 mL) was added Et_3_N (19 µL, 0.14 mmol) at room temperature, and the mixture was stirred overnight. The mixture was concentrated *in* *vacuo*. The residue was purified by silica gel column chromatography (hexane/acetone = 2:1) to give **8** as an oil (37 mg, 95%): [α]^23^_D_ −11.5 (*c* 1.00, CHCl_3_); IR (film) 3523, 1731, 1601, 1263 cm^−1^; ^1^H-NMR (400 MHz) *δ* 1.05 (3H, m, cyclohexyl), 1.48 (6H, m, cyclohexyl), 1.77 (1H, m, cyclohexyl), 2.41 (1H, bs, OH), 3.68 (4H, m, cyclohexyl, H-5,6a,6b), 4.38 (1H, d, *J* = 8.0 Hz, H-1), 5.37 (2H, bt, H-2,4), 5.82 (1H, t, *J* = 9.6 Hz, H-3), 7.18 (3H, m, Ar), 7.31 (4H, m, Ar), 7.41 (2H, m, Ar), 7.74 (2H, m, Ar), 7.85 (4H, m, Ar); ^13^C-NMR (100 MHz) δ 23.4 (cyclohexyl), 23.6 (cyclohexyl), 25.4 (cyclohexyl), 31.5 (cyclohexyl), 33.2 (cyclohexyl), 61.5 (C-6), 69.7 (C-4,4',6'), 72.1 (C-2,3,3'), 72.9 (C-2',5'), 74.5 (C-5), 78.1 (cyclohexyl), 99.6 (C-1,1'), 128.2 (Ar), 128.3 (Ar), 128.5 (Ar), 128.6 (Ar), 128.9 (Ar), 129.5 (Ar), 129.6 (Ar), 129.7 (Ar), 129.9 (Ar), 133.1 (Ar), 133.2 (Ar), 133.6 (Ar), 164.9 (C=O), 165.8 (C=O), 165.9 (C=O). ESI-MS: calcd for C_33_H_34_O_9_Na 597.2101 (M+Na)^+^, found *m/z* 597.2112.

Cyclohexyl 2,3,4-tri-O-benzoyl-β-d-glucopyranosyl-(1→6)-2,3,4-tri-O-benzoyl-6-O-*9-fluorenyl-methyloxycarboxyl*-β-d-glucopyranoside (**9**). A solution of cyclohexanol in 1,2-DCE (10 mL, 0.1 M) containing 4Å MS and Bu_4_NClO_4_ (3.42 g, 1 M) as a supporting salt, was electrolyzed by constant current electrolysis (C.C.E., 6 mA/cm^2^) at 40 °C, using a glassy carbon plate (1.5 cm × 1.5 cm) as an anode and a Pt plate (1.8 cm × 1.8 cm) as a cathode. The reaction mixture (0.6 mL, 0.1 M EGA solution, 3 equiv.) was added to a solution of **2a** (31 mg, 0.032 mmol) and **8** (37 mg, 0.064 mmol), and MS 4A in 1,2-DCE (0.5 mL). After being stirred 20 min, the mixture was filtered through a Celite pad, and the filtrate was washed with sat. aq. NaHCO_3_. The organic extract was dried (Na_2_SO_4_), and concentrated *in vacuo*. The residue was purified by preparative TLC (hexane/acetone = 2:1) to give **9** as an oil (16 mg, 38%): [α]^23^_D_ −9.8 (c 1.00, CHCl_3_); IR (film) 1734, 1601, 1261 cm^−1^; ^1^H-NMR (400 MHz) δ 1.41 (10H, m, cyclohexyl), 3.62 (1H, m, cyclohexyl), 4.00 (4H, m, Fmoc, H-5,5'), 4.29 (5H, m, H-6a,6b,6a',6b'), 4.78 (1H, d, *J* = 7.8 Hz, H-1'), 5.04 (1H, d, *J* = 7.8 Hz, H-1), 5.34 (1H, t, *J* = 9.8 Hz, H-4'), 5.41 (1H, dd, *J* = 9.8, 7.8 Hz, H-2'), 5.48 (1H, dd, *J* = 9.8, 7.8 Hz, H-2), 5.52 (1H, t, *J* = 9.8 Hz, H-4), 5.80 (1H, t, *J* = 9.8 Hz, H-3'), 5.83 (1H, t, *J* = 9.8 Hz, H-3), 7.41 (22H, m, Ar), 7.62 (2H, m, Ar), 7.78 (6H, m, Ar), 7.92 (8H, m, Ar); ^13^C-NMR (100 MHz) δ 22.9 (cyclohexyl), 23.2 (cyclohexyl), 25.4 (cyclohexyl), 31.2 (cyclohexyl), 32.9 (cyclohexyl), 46.6 (Fmoc), 66.0 (C-6), 68.3 (C-6'), 69.3(C-4), 69.9 (C-4'), 70.3 (C-3), 71.7 (C-2,3'), 71.9 (C-2'), 72.1 C-5'), 72.8 (C-5), 72.9 (cyclohexyl), 74.1 (FmocCH_2_), 99.4 (C-1'), 100.8 (C-1), 119.9 (Ar), 125.3 (Ar), 125.4 (Ar), 127.2 (Ar), 127.8 (Ar), 128.2 (Ar), 128.3 (Ar), 128.4 (Ar), 128.7 (Ar), 128.9 (Ar), 129.3 (Ar), 129.5 (Ar), 129.6 (Ar), 129.7 (Ar), 129.8 (Ar), 133.0 (Ar), 133.1 (Ar), 133.2 (Ar), 133.4 (Ar), 133.5 (Ar), 141.2 (Ar), 143.3 (Ar), 143.4 (Ar), 154.8 (C=O), 164.9 (C=O), 165.1 (C=O), 165.2 (C=O), 165.4 (C=O), 165.6 (C=O), 165.7 (C=O). ESI-MS: calcd for C_75_H_66_O_19_Na 1293.4096 (M+Na)^+^, found m/z 1293.4078.

*Phenyl 2,3,4-tri-O-benzoyl-6-O-9-fluorenylmethyloxycarboxyl-β-d-glucopyranosyl-(1→6)-2,3,4-tri-O-benzoyl-1-thio-β-d-glucopyranoside* (**11**). A solution of cyclohexanol in 1,2-DCE (10 mL, 0.1 M) containing 4Å MS and Bu_4_NClO_4_ (3.42 g, 1 M) as a supporting salt was electrolyzed by constant current electrolysis (C.C.E., 6 mA/cm^2^) at 40 °C, using a glassy carbon plate (1.5 cm × 1.5 cm) as an anode and a Pt plate (1.8 cm × 1.8 cm) as a cathode. After the electrolysis, the reaction mixture (1.3 mL, 0.1 M EGA solution, 3 equiv.) was added to a solution of **2a** (34 mg, 0.036 mmol), **10** (42 mg, 0.072 mmol), m and 4Å MS in 1,2-DCE (0.5 mL). After being stirred for 20 min, the reaction mixture was filtered through a Celite pad, and the filtrate was washed with sat. aq. NaHCO_3_. The organic extracts were dried (Na_2_SO_4_), and concentrated *in vacuo*. The residue was purified by preparative TLC (hexane/acetone = 2:1) to give **11** as an oil (29 mg, 63%): [α]^23^_D_ +8.9 (c 1.00, CHCl_3_); IR (film) 1733, 1601, 1261 cm^−1^; ^1^H-NMR (400 MHz) δ 3.99 (4H, m, Fmoc, H-5,5'), 4.24 (1H, m, Fmoc), 4.38 (4H, m, H-6a,6b,6a',6b'), 4.92 (1H, d, *J* = 9.8 Hz, H-1'), 4.97 (1H, d, *J* = 8.0 Hz, H-1), 5.31 (1H, t, *J* = 9.8 Hz, H-4'), 5.34 (1H, t, *J* = 9.8 Hz, H-3'), 5.51 (2H, m, H-2,4), 5.81 (1H, t, *J* = 9.8 Hz, H-3'), 5.85 (1H, t, *J* = 9.8 Hz, H-3), 7.23 (2H, m, Ar), 7.39 (25H, m, Ar), 7.61 (2H, m, Ar), 7.74 (4H, m, Ar), 7.85 (4H, m, Ar), 7.93 (6H, m, Ar); ^13^C-NMR (100 MHz) δ 46.6 (Fmoc), 65.9 (C-6), 68.4 (C-6'), 69.4 (C-4'), 69.5 (C-4'), 70.2 (C-3'), 70.4 (C-3), 71.6 (C-2), 72.0 (C-2'), 72.8 (C-5'),74.0 (FmocCH_2_), 78.3 (C-5), 85.9 (C-1'), 101.0 (C-1), 119.9 (Ar), 125.3 (Ar), 125.4 (Ar), 127.2 (Ar), 128.2 (Ar), 128.3 (Ar), 128.4 (Ar), 128.5 (Ar), 128.6 (Ar), 128.7 (Ar), 128.8 (Ar), 129.1 (Ar), 129.2 (Ar), 129.7 (Ar), 129.8 (Ar), 131.7 (Ar), 133.2 (Ar), 133.4 (Ar), 133.5 (Ar), 141.2 (Ar), 141.3 (Ar), 143.3 (Ar), 143.4 (Ar), 154.8 (C=O), 164.9 (C=O), 165.1 (C=O), 165.2 (C=O), 165.3 (C=O), 165.6 (C=O), 165.7 (C=O). ESI-MS: calcd for C_75_H_60_O_18_NaS 1303.3398 (M+Na)^+^, found *m/z* 1303.3385.

*Phenyl 2,3,4-tri-O-benzoyl-6-O-9-fluorenylmethyloxycarboxyl-β-d-glucopyranosyl-(1→6)-2,3,4-tri-O-benzoyl-β-d-glucopyranosyl-(1→6)-2,3,4-tri-O-benzoyl-1-thio-β-d-glucopyranoside* (**12**). To a solution of **11** (29 mg, 0.022 mmol) in pyridine (0.5 mL) was added Et_3_N (6.3 µL, 0.045 mmol) at room temperature. After being stirred overnight, the mixture was concentrated *in vacuo*. The residue was purified by silica gel column chromatography (hexane/acetone = 2:1) to give an alcohol as an oil (19 mg, 82%): [α]^23^_D_ −2.1 (c 1.00, CHCl_3_); IR (film) 3062, 1731, 1601, 1262 cm^−1^; ^1^H-NMR (400 MHz) δ 2.81 (1H, dd, *J* = 8.0, 5.6 Hz, OH), 3.60 (1H, m, H-5), 3.74 (2H, m, H-5',6a'), 3.95 (1H, dd, *J* = 10.5, 5.6 Hz, H-6b'), 4.05 (2H, m, H-6a,6b), 4.92 (2H, bt, H-1,1'), 5.23 (1H, t, *J* = 9.6 Hz, H-4'), 5.40 (3H, m, H-2,2'), 5.81 (1H, t, *J* = 9.6 Hz, H-3'), 5.85 (1H, t, *J* = 9.6 Hz, H-3), 7.38 (19H, m, Ar), 7.53 (4H, m, Ar), 7.76 (2H, m, Ar), 7.81 (2H, m, Ar), 7.93 (8H, m, Ar); ^13^C-NMR (100 MHz) δ 61.2 (C-6), 67.9 (C-6'), 69.4 (C-4'), 69.9 (C-4), 70.4 (C-3'), 71.5 (C-3), 72.8 (C-2), 73.9 (C-2'), 74.6 (C-5'), 77.8 (C-5), 86.0 (C-1'), 100.5 (C-1), 128.2 (Ar), 128.3 (Ar), 128.4 (Ar), 128.5 (Ar), 128.7 (Ar), 128.8 (Ar), 129.1 (Ar), 129.2 (Ar), 129.7 (Ar), 129.8 (Ar), 129.9 (Ar), 131.5 (Ar), 133.2 (Ar), 133.3 (Ar), 133.5 (Ar), 133.6 (Ar), 164.9 (C=O), 165.1 (C=O), 165.6 (C=O), 165.7 (C=O), 165.9 (C=O). ESI-MS: calcd for C_60_H_50_O_16_NaS 1081.2717 (M+Na)^+^, found *m/z* 1081.2732.

A solution of cyclohexanol in 1,2-DCE (10 mL, 0.1 M) containing 4Å MS and Bu_4_NClO_4_ (3.42 g, 1 M) as a supporting salt, was electrolyzed by constant current electrolysis (C.C.E., 6 mA/cm^2^) at 40 °C, using a glassy carbon plate (1.5 cm × 1.5 cm) as an anode and a Pt plate (1.8 cm × 1.8 cm) as a cathode. The reaction mixture (0.6 mL, 0.1 M EGA solution, 3 equiv.) was added to a solution of **2a** (37 mg, 0.038 mmol), the alcohol (20 mg, 0.019 mmol), and 4Å MS in 1,2-DCE (0.5 mL). After being stirred at 40 °C for 20 min, the reaction mixture was filtered through a Celite pad, and the filtrate was washed with saturated aqueous NaHCO_3_. The organic extracts were dried (Na_2_SO_4_), and concentrated *in vacuo*. The residue was purified by preparative TLC (Et_2_O/hexane = 2:1) to give **12** as a clear oil (4.1 mg, 12%): [α]^23^_D_ −5.4 (*c* 1.00, CHCl_3_); IR (film) 1733, 1601, 1261 cm^−1^; ^1^H-NMR (400 MHz) δ 3.62 (1H, dd, *J* = 11.2, 5.0 Hz, H-6a), 3.84 (3H, m, Fmoc, H-5',6), 4.01 (2H, m, H-5,5''), 4.31 (6H, m, H-6a,6b,6a',6b'), 4.61 (1H, d, *J* = 8.0 Hz, H-1'), 4.99 (1H, d, *J* = 10.0 Hz, H-1''), 5.08 (1H, d, *J* = 8.0 Hz, H-1), 5.10 (1H, t, *J* = 10.0 Hz, H-4), 5.21 (1H, dd, *J* = 9.8, 8.0 Hz, H-2''), 5.51 (4H, m, H-2,2',4',4''), 5.68 (1H, t, *J* = 9.8 Hz, H-3'), 5.84 (1H, t, *J* = 9.8 Hz, H-3''), 6.06 (1H, t, *J* = 9.8 Hz, H-3), 7.18 (2H, m, Ar), 7.36 (34H, m, Ar), 7.58 (2H, m, Ar), 7.76 (2H, m, Ar), 7.92 (14H, m, Ar); ^13^C-NMR (100 MHz) δ 46.6 (Fmoc), 66.2 (C-6), 68.3 (C-6''), 69.4 (C-6'), 69.7 (C-4''), 70.1 (C-4), 70.2 (C-4'), 70.5 (C-3''), 71.7 (C-3), 71.9 (C-2,2',3'), 72.0 (C-2''), 72.6 (C-5'), 72.7 (C-5''), 73.9 (C-5), 74.1 ((FmocCH_2_), 86.3 (C-1''), 100.5 (C-1), 101.2 (C-1'), 119.9 (Ar), 120.0 (Ar), 125.3 (Ar), 125.4 (Ar), 127.2 (Ar), 127.8 (Ar), 128.1 (Ar), 128.2 (Ar), 128.3 (Ar), 128.4 (Ar), 128.5 (Ar), 128.6 (Ar), 128.7 (Ar), 128.8 (Ar), 129.0 (Ar), 129.1 (Ar), 129.3 (Ar), 129.4 (Ar), 129.7 (Ar), 129.8 (Ar), 130.0 (Ar), 132.1 (Ar), 132.8 (Ar), 133.0 (Ar), 133.2 (Ar), 133.4 (Ar), 141.2 (Ar), 143.3 (Ar), 143.4 (Ar), 154.8 (C=O), 164.9 (C=O), 165.2 (C=O), 165.3 (C=O), 165.7 (C=O). ESI-MS: calcd for C_102_H_82_O_26_NaS 1777.4713 (M+Na)^+^, found *m/z* 1777.4736.

## 4. Conclusions

The orthoester **2a** bearing the selectively removable Fmoc group at the C-6 position was synthesized, and its utility as a glycosyl donor and acceptor after deprotection of the Fmoc group was demonstrated.

## References

[1] Gantt R.W., Peltier-Pain P., Thorson J.S. (2011). Enzymatic methods for glyco(diversification/randomization) of drugs and small molecules. Nat. Prod. Rep..

[2] Toshima K. (2013). Chemical biology based on target-selective degradation of proteins and carbohydrates using light-activatable organic molecules. Mol. Biosyst..

[3] Toshima K., Tatsuta K. (1993). Recent Progress in *O*-Glycosylation Methods and Its Application to Natural Products Synthesis. Chem. Rev..

[4] Jacobsson M., Malmberg J., Ellervik U. (2006). Aromatic *O*-glycosylation. Carbohydr. Res..

[5] Stallforth P., Lepenies B., Adibekian A., Seeberger P.H. (2009). Carbohydrates: A frontier in medicinal chemistry. J. Med. Chem..

[6] Liptak A., Borbas A., Bajza I., Kamering J.P., Boons G.-J., Lee Y.C., Suzuki A., Taniguchi N., Vorangen A.G.J. (2007). Protective Group Manipulation in Carbohydrate Synthesis. Comprehensive Glycosciences.

[7] Kochetkov N.K., Bochkov A.F., Sokolovskaya T.A., Snyatkova V.J. (1971). Modifications of the orthoester method of glycosylation. Carbohydr. Res..

[8] Bochkov A.F., Kochetkov N.K. (1975). A new approach to the synthesis of oligosaccharides. Carbohydr. Res..

[9] Bataneli V.I., Ovchinnikov M.V., Backinowsky L.V., Kochetkov N.K. (1979). Glycosylation by 1,2-*O*-cyanothylidene derivatives of carbohydrates. Carbohydr. Res..

[10] Kochetkov N.K., Nepogod’ev S.A., Backinowsky L.V. (1990). Synthesis of cyclo-[(1–6)-β-d-galactofurano]-oligosaccharides. Tetrahedron.

[11] Backinowaky L.V., Tavetkov Y.E., Balan N.F., Byramova N.E., Kochetkov N.K. (1980). Synthesis of 1,2-*trans*-disaccharides *via* sugar thio-orthoesters. Carbohydr. Res..

[12] Kunz H., Harreus A. (1982). Glycosidsynthese mit 2,3,4,6-tetra-*O*-pivaloyl-α-d-glucopyrancsylbromid. Liebigs Ann. Chem..

[13] Kunz H., Pfrengle W. (1986). Effective 1,2-*trans*-glycosylation of complex alcohols and phenols using the oximateorthoester of *O*-pivaloyl glucopyranose. Chem. Commun..

[14] Kawa K., Saitoh T., Kaji E., Nishiyama S. (2013). Development of glycosylation using the glucopyranose 1,2-orthobenzoate under electrochemical conditions. Org. Lett..

[B15-molecules-19-02602] 15.EGA was discussed in several papers [16,17,18,19,20].Upon comparison of methods using EGA, the case using EGA after preparation by anodic oxidation, was preferable to the simultaneous EGA-preparation and glycosylation [14]. A lower oxidation potential of cyclohexanol compared with the orthoester derivative in the reaction mixture, was effectively used for the abstraction of proton.

[16] Uneyama K., Steckhan E. (1987). Electrochemistry 1.

[17] Torii S. (1985). Electroorganic Synthesis, Part 1, Oxidation Methods, Applications.

[18] Uneyama K., Ishimura A., Torii S. (1985). Electrogenerated acid-catalyzed cyclization of isoprenoids. Bull. Chem. Soc. Jpn..

[19] Nielsen M.F., Shäfer H.J. (2004). Encyclopedia of Electrochemistry.

[20] Matsuo K., Shimazaki H., Sanada T., Shimada K., Hagiwara S., Suga S., Kashimura S., Yoshida J. (2013). Electrogenerated acid (EGA)-catalyzed addition of diaryl disulfides to carbon-carbon multiple bonds. Chem. Lett..

[B21-molecules-19-02602] 21.When the glycosylation using Ag_2_CO_3_, the yield of **2a** was reduced to 17%.

[B22-molecules-19-02602] 22.The reaction condition has not yet optimized.

